# Effect of Transcranial Direct Current Stimulation Combined with Rehabilitation on Arm and Hand Function in Stroke Patients: A Systematic Review and Meta-Analysis

**DOI:** 10.3390/healthcare9121705

**Published:** 2021-12-08

**Authors:** Joo-Hyun Lee, Yu-Jin Jeun, Hae Yean Park, Young-Jin Jung

**Affiliations:** 1 Department of Occupational Therapy, Baekseok University, Cheonan 31065, Korea; otlove@bu.ac.kr; 2 Department of ICT Convergence, The Graduate School, Soonchunhyang University, Asan 31538, Korea; eie0305@naver.com; 3 Department of Occupational Therapy, College of Software and Digital Healthcare Convergence, Yonsei University, Wonju 26493, Korea; haepark@yonsei.ac.kr; 4 School of Healthcare and Biomedical Engineering, Chonnam National University, Yeosu 59626, Korea

**Keywords:** arm and hand function, meta-analysis, rehabilitation, stroke, systematic review, transcranial direct current stimulation

## Abstract

Transcranial direct current stimulation (tDCS) is a noninvasive brain stimulation technique that may enhance motor recovery after stroke. We performed a systematic review and meta-analysis to assess the efficacy of tDCS combined with rehabilitation on arm and hand function after stroke. Electronic databases were searched from their inception to September 2021. We performed a systematic review of selected randomized controlled trials, and methodological qualities were measured using the PEDro (Physiotherapy Evidence Database) scale. We calculated the standardized mean difference for effect size using the Comprehensive Meta-Analysis 3.0 software. We selected 28 studies for the systematic review and 20 studies for the meta-analysis. The overall effect size was 0.480 (95% CI [0.307; 0.653], *p* < 0.05), indicating a moderate effect size of tDCS combined with rehabilitation for upper extremity function in stroke survivors. The tDCS with occupational therapy/physical therapy (0.696; 95% CI [0.390; 1.003], *p* < 0.05) or virtual reality therapy (0.510; 95% CI [0.111; 0.909], *p* < 0.05) was also significantly more effective than other treatments. This meta-analysis of 20 randomized controlled trials provides further evidence that tDCS combined with rehabilitation, especially occupational therapy/physical therapy and virtual reality therapy, may benefit upper extremity function of the paretic upper limb in stroke patients.

## 1. Introduction

Non-invasive brain stimulation techniques, such as repetitive transcranial magnetic stimulation (rTMS) and transcranial direct current stimulation (tDCS), have been used in recent years not only to enhance neural plasticity, but also to improve motor function in the context of post-stroke rehabilitation [1]. Although rTMS and tDCS are useful techniques for painless and non-invasive stimulation of the human brain, tDCS is more suitable as a therapeutic tool as it can be applied more easily than rTMS [2]. There are three tDCS methods: (i) anodal tDCS; (ii) cathodal tDCS as unilateral tDCS, which modulates cortical excitability in a polarity-dependent manner at the stimulated primary motor cortex (M1); and (iii) bihemispheric tDCS, which induces both up- and down- regulation of M1 excitability by applying anodal stimulation to M1 in one hemisphere and cathodal stimulation to that in the other hemisphere [3]. Through electrodes applied to the scalp, these stimulation approaches can modulate excitable and inhibitory neuronal networks in both affected and non-affected hemispheres to improve motor function on the paretic side in stroke patients [4].

Paralysis of the affected upper extremity interferes with the independent daily life of stroke patients; it induces abnormal muscle tone, weakness, and coordination problems. Recent studies on the application of tDCS combined with rehabilitation have shown positive results regarding arm and hand recovery in stroke patients. These results indicate that tDCS combined with rehabilitation can maximize the effects of rehabilitation training by removing the imbalance in transcallosal inhibition after subcortical stroke [5]. A study using tDCS in combination with occupational therapy in patients with chronic stroke also demonstrated that fine motor skill deficits can be significantly improved when these interventions are applied simultaneously [6]. However, to date, there have been no meta-analyses providing clearly integrated information regarding the characteristics of tDCS and rehabilitation when applied as a combined intervention. Similarly, no meta-analysis has previously assessed the efficacy of combination therapy for stroke patients.

A systematic review regarding the efficacy of anodal tDCS for upper limb motor recovery reported that anodal tDCS may improve motor function in patients with chronic stroke. Another published systematic review [7], which included 26 studies with 754 participants, compared the effects of active tDCS (anodal, cathodal, or dual), sham tDCS, and physical rehabilitation with respect to the improvement of activities of daily living capacity and arm function. The results indicated that cathodal tDCS applied to the non-lesioned brain area was significantly more effective than other stimulation or rehabilitation techniques in improving the activities of daily living capacity in stroke patients. However, these reviews did not focus on a combination of tDCS and rehabilitation. In particular, in contrast with cathodal tDCS, the application of anodal tDCS, dual tDCS, and sham tDCS had no significant effects on the activities of daily living capacity in stroke patients [7].

Thus, a systematic investigation of the effectiveness of tDCS combined with rehabilitation is required to clarify its effects on stroke rehabilitation. Meta-analysis is a useful method for quantitatively synthesizing data from clinical trials; it offers greater statistical power, and is more reliable and objective than a single analysis [8].

The purpose of our systematic review was to obtain an overview of the available scientific evidence to determine whether tDCS combined with rehabilitation has any effect on upper extremity function of the paretic side after stroke.

## 2. Materials and Methods

We performed a systematic literature review to analyze the effectiveness of tDCS combined with rehabilitation for upper extremity function recovery in stroke patients.

The inclusion criteria for the studies are as follows: (1) a randomized control trial (RCT) or pilot-RCT study design; (2) a patient population of adults >18 years old who had experienced any type of stroke (ischemic or hemorrhage); (3) anodal, cathodal, and dual tDCS combined with therapeutic interventions that were related to rehabilitation such as occupational therapy, physical therapy, robot-assisted therapy, virtual reality therapy, and constraint-induced movement therapy; and (4) outcome measures that were classified as functional ability or recovery of the upper extremity (shoulder, hand, and arm) measured by a validated specific assessment technique, such as the Action Research Arm Test [9] or Fugl-Meyer Assessment [10]. The experimental group comprised patients who underwent tDCS combined with rehabilitation, and the control group included patients who underwent sham tDCS combined with rehabilitation or those who underwent only rehabilitation.

We searched the following electronic databases for articles published until September 2021: Medline (from 2009), Embase (from 2011), Cochrane register of controlled clinical trials (from 2014), Scopus (from 2006), and other sources (Psychlnfo, Book) for identifying additional records. The search strategies are provided in Appendix A. Our research protocol follows the Preferred Reporting Items for Systematic Reviews and Meta-Analyses guidelines. We registered this analysis in the PROSPERO database (ID: CRD42018109085).

The methodological quality of the selected studies was assessed using the Physiotherapy Evidence Database (PEDro) scale. The PEDro scale was originally developed to measure the methodological quality of clinical trials [11] and consists of 11 items that measure the external validity (criterion 1), internal validity (criteria 2–9), and statistical information (criteria 10–11) of a given trial. The range of scores pertaining to internal validity and statistical information is 0–10, and the following cutoff points are used: 9–10, excellent; 6–8, good; 4–5, fair; and below 4, poor [12].

The effect size and publication bias of the selected studies were analyzed using Comprehensive Meta-Analysis version 3.0 (Biostat; Englewood, NJ, USA). An effect size was defined as the difference between the means of the experimental and control groups after a given intervention was divided by the standard deviation of the control group. We analyzed the mean difference, standardized mean difference, and 95% confidence interval (CI) to measure effect sizes, which were then used to compare the effects of tDCS combined with other rehabilitations with those of control interventions. The effect sizes of this meta-analysis were reported as small (d > 0.2), moderate (d > 0.5), and large (d > 0.8), as described in a previous study [13]. A positive effect size indicates a better effect with tDCS combined with other rehabilitations compared to rehabilitation alone, while a negative effect size suggests that rehabilitation alone had better effects than combination training.

It is important to assess the extent of heterogeneity among the studies used in a meta-analysis. The extent of heterogeneity can be measured by a heterogeneity test that indicates the variance in each study using Q-statistic and I-squared values. When the Q-value is significant (*p* < 0.1), heterogeneity is present among the studies [14]. The extent of heterogeneity can also be interpreted by I-squared values, ranging from absent (0) to low (25), medium (50), or high (75) [15]. If the effects among studies are significantly heterogeneous, a random-effects model can be used to estimate the overall effect in a meta-analysis. On the other hand, if the effects among studies are significantly homogeneous, a fixed effect model can be used [16].

Publication bias refers to the distortion of the results of a meta-analysis that can occur by including more studies with positive results than those with negative results, because studies with less significant or negative results are less likely to be published [17]. The existence of publication bias was assessed using a funnel plot and Egger’s regression. A funnel plot is a graph with the effect size on the *x*-axis and standard error on the *y*-axis. If the pattern of a given funnel plot becomes asymmetrical, with more one-sided results, it means that publication bias is present. The Egger’s regression intercept test also detects publication bias by measuring the intercept from regression between the effect size and standard error. When the regression is insignificant (*p* > 0.05), publication bias is considered absent [18].

## 3. Results

**Figure 1:**
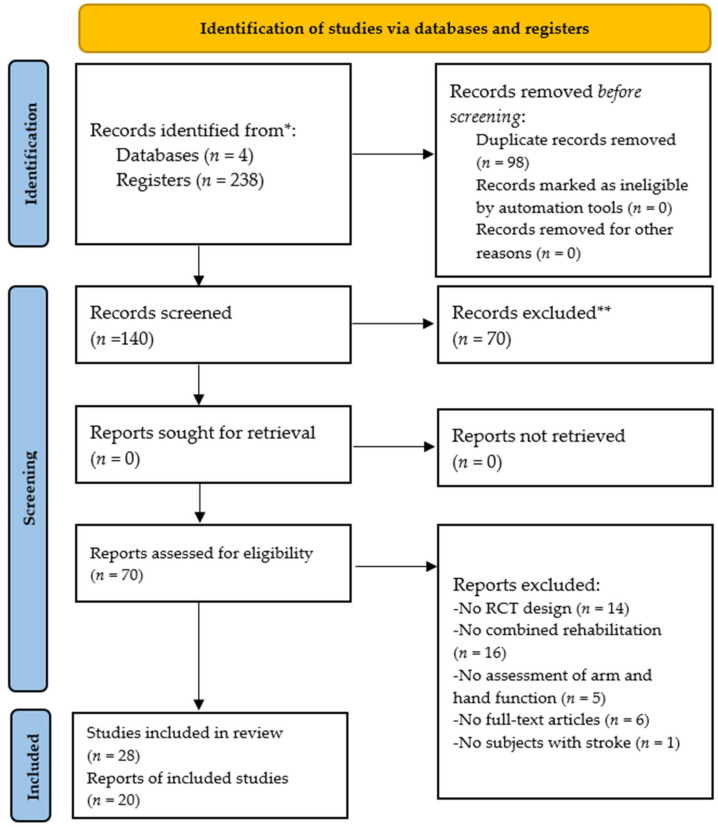
Preferred Reporting Items for Systematic Reviews and Meta-Analyses (PRISMA) 2020 flow diagram of study selection. *, record identification; **, record exclusion. RCT, randomized controlled trial.

We identified 238 articles from four databases (Medline, Embase, Cochrane register of controlled clinical trials, and Scopus) and screened 140 articles after removing duplicate articles. Seventy articles were removed after reviewing the title and abstract. Of the 70 articles selected for full-text review, 42 were excluded because they failed to meet certain inclusion criteria, such as stroke diagnoses, use of combined intervention, or study design (e.g., review articles). Finally, we selected 28 studies for qualitative synthesis, including 20 studies used for quantitative meta-analysis (Figure 1).

The total number of patients in the 20 RCTs was 818, including 453 in the experimental group and 365 in the control group (mean age range: 49.3–75.3 years). The mean time of stroke onset ranged from 5.9 days (acute phase: 0–7 days) to 56.6 months (chronic phase: >6 months). The rehabilitation used in combination with tDCS was occupational therapy/physical therapy in eight studies, robot-assisted therapy in six studies, virtual reality therapy in six studies, and constraint-induced movement therapy in three studies. The duration of rehabilitation training varied among the studies from 20 min to 6 h per day and from five-day to eight-week periods. The most common analytical tools used to assess upper extremity function or recovery in stroke patients were the upper extremity Fugl-Meyer Test, Wolf Motor Function Test, Box and Block Test, and Action Research Arm Test (Table 1).

The type of tDCS used was anodal in nine studies, dual in eleven studies, cathodal in three studies, and anodal and cathodal in four studies; one study provided limited information regarding tDCS. Anodal tDCS was applied with the anode placed over the M1 of the affected hemisphere and the cathode over the contralesional supraorbital region. For cathodal tDCS, the cathode was placed over the contralesional motor region, and the anode was placed on the supraorbital region of the affected hemisphere. The average amount of applied current ranged from 1–2 mA (milliampere). A current of 20 mA was applied only in one study. The delivery duration ranged from 9–30 min (Table 2). The mean (standard deviation) PEDro score across all 28 studies was 7.7 (1.7), with a range from 4 (fair) to 11 (excellent) points. These results indicate that the quality of all reviewed studies was sufficient for the meta-analysis (Table 1).

In this meta-analysis, we examined 20 studies comparing the effects of tDCS combined with rehabilitation to those of sham tDCS with rehabilitation or rehabilitation only in stroke patients. Table 3 presents the effect size, overall Q-squared, and I-squared values. The overall effect size for the effectiveness of tDCS combined with rehabilitation was 0.480 (95% CI [0.307, 0.653], *p* < 0.05), which indicates that the combination of tDCS and rehabilitation had a higher value than sham tDCS with rehabilitation or rehabilitation only [13]. We used a fixed effect model to assess the variation within and between studies because the p-value of the Q-value was greater than 0.05 in the heterogeneity test, and the I-squared value of 0.000 suggested the absence of heterogeneity (Table 3).

**Figure 2:**
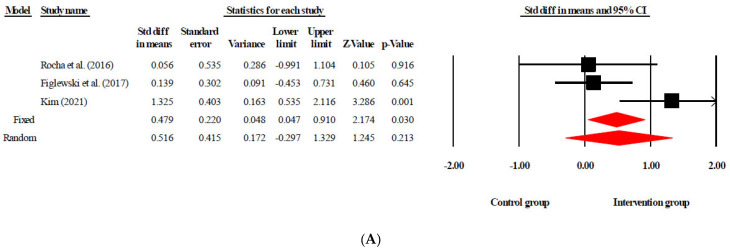

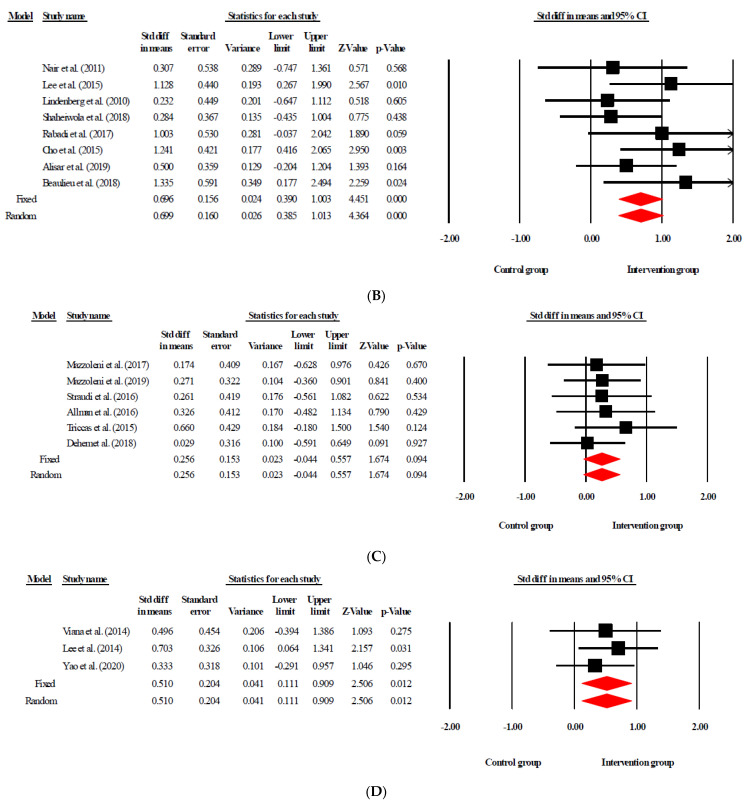
Forest plot showing the effect sizes for transcranial direct current stimulation combined with rehabilitation in stroke patients. (**A**) Constraint-induced movement therapy. (**B**) Occupational therapy/physical therapy. (**C**) Robot therapy. (**D**) Virtual reality therapy. Std diff: standard difference.

In total, eight studies were included in the occupational/physical therapy group. The effect size of tDCS combined with occupational therapy/physical therapy for stroke patients was 0.696 (95% CI [0.390, 1.003], *p* < 0.05), which is interpreted as a medium effect size. Three studies were included in the virtual reality therapy group, and the effect size of tDCS combined with virtual reality therapy was 0.510 (95% CI [0.111, 0.909], *p* < 0.05), which is interpreted as a medium effect size. Next, three studies were included in the constraint-induced movement therapy group. The effect size of tDCS combined with CIMT was 0.479 (95% CI [0.047, 0.910], *p* < 0.05), which is interpreted as close to the median effect size. The remaining six studies were included in the robot-assisted therapy group, and the effect size of tDCS combined with robot-assisted therapy was 0.256 (95% CI [–0.044, 0.557], *p* > 0.05), which is interpreted as a small effect size (Figure 2).

**Figure 3:**
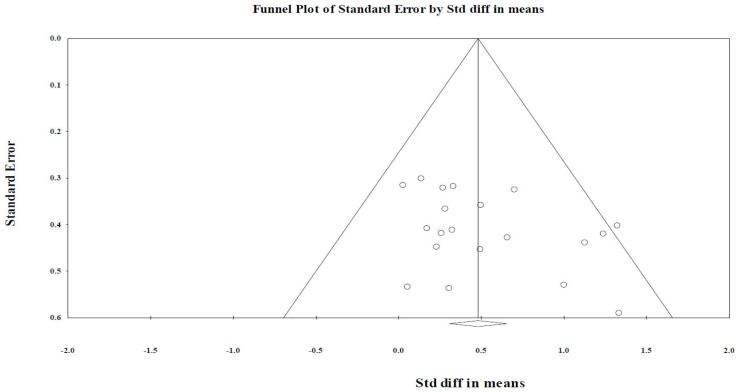
Funnel plot for publication bias. Std diff: standard difference.

Funnel plots of effect size against precision were used to investigate the presence of publication bias. The funnel plot was symmetrical across the center of the mean vertical axes of the funnel plot (Figure 3).

## 4. Discussion

We aimed to compare the efficacy of tDCS with rehabilitation and that of sham tDCS with rehabilitation or rehabilitation alone using a systematic review and meta-analysis. This systematic review included 28 randomized controlled trials with 818 stroke patients who underwent occupational therapy/physical therapy, robot-assisted therapy, virtual reality therapy, and constraint-induced movement therapy. The upper extremity Fugl-Meyer Test, Wolf Motor Function Test, Box and Block Test, and Action Research Arm Test were used for the measurement of upper arm and hand movement function.

The analytic results of the 20 articles included in the meta-analysis revealed a significant overall effect size of tDCS combined with rehabilitation. Although conflicting results were found in a previous network meta-analysis, in that stroke patients showed no significant improvement in arm function with only tDCS [7], our findings suggest that tDCS combined with rehabilitation was more effective for upper extremity function recovery in stroke patients than sham tDCS with rehabilitation or rehabilitation only. Notably, our findings corroborate the findings of another meta-analysis published previously [47]. One possible reason for these findings is use-dependent plasticity changes, which occur in a multiregional brain network when non-invasive brain stimulation is applied in combination with rehabilitation [48]. In other words, the effectiveness of upper extremity rehabilitation can be increased substantially when used together with non-invasive brain stimulation. However, further studies are required to assess the difference in effect according to the degree of damage and onset of stroke.

Among the four types of rehabilitation, the effect size of tDCS in combination with occupational therapy/physical therapy or virtual reality therapy was greater than that of robot-assisted therapy or constraint-induced movement therapy in stroke patients. The occupational therapy/physical therapy used in eight studies was administered by trained therapists, and included proprioceptive neuromuscular facilitative approaches, functional motor tasks, goal-directed activities, mirror therapy, and task-oriented movements with functional electrical stimulation [23,30,33,37,38,46]. The virtual reality therapy used in three studies were therapeutic approaches that allowed patients to interact with tools such as computer games, programs for motor training, and virtual and robotic systems, thereby minimizing assistance from therapists [20,31,34,42,43,44]. Furthermore, the constraint-induced movement therapy used in three studies was administered to patients during their daily activities, and they were provided instructions regarding proper technique by the therapists [25,39].

The results of this meta-analysis suggest that tDCS combined with interventions provided by therapists had a greater effect on upper extremity function in stroke patients than interventions provided primarily by equipment, highlighting the important role of therapists in rehabilitation. These results have great implications in terms of the clinical application of tDCS combined with rehabilitation. tDCS in combination with occupational therapy/physical therapy or virtual reality therapy had a larger effect size than with robot-assisted therapy or constraint-induced movement therapy; however, this does not mean that tDCS in combination with robot-assisted therapy or constraint-induced movement therapy should not be considered. The reason is that the period and frequency of the four types of rehabilitation applied together with tDCS as well as the fact that the patient conditions were different should be considered. Thus, each clinician should select applicable rehabilitation treatments along with their own treatment environment and tDCS by referring to the results of this study in the rehabilitation setting. Moreover, they need to consider several factors before administering rehabilitation treatments. The efficacy of equipment systems for robot-assisted therapy may be somewhat influenced by the lack of computer skills of some therapists or the lack of interaction between therapists and patients [49]. Additionally, most occupational therapy/physical therapy involves performing tasks based on activities of daily living at home rather than in a clinical context. Therefore, it is important to actively provide interventions using daily living tasks for stroke patients by relying on caregivers, therapists, or the patients themselves in a home-like setting, rather than in simulations.

The funnel plot of effect size to identify publication bias in the 20 selected studies had a symmetrical funnel shape centering around the mean effect size plot, and the Egger’s regression intercept had a *p*-value lesser than 0.05. Thus, the studies selected for this meta-analysis had no significant publication bias [50].

Our meta-analysis had some limitations. First, the control group in our study included patients who underwent sham tDCS, solely tDCS, or rehabilitation. This negatively affects the interpretability of the results, and placebo effects of real tDCS cannot be excluded. Future studies should focus on comparisons with a control group receiving sham tDCS. Second, we focused on effective interventions combined with tDCS. It is necessary to include different outcome measures such as the characteristics of stroke patients, types of applied tDCS, and attachment area of the electrodes during tDCS. Third, constraint-induced movement therapy and virtual reality therapy were used in fewer studies compared with other rehabilitation types; this may have influenced the effect size of the meta-analysis.

## 5. Conclusions

Despite the abovementioned limitations, this meta-analysis suggests that tDCS combined with rehabilitation can improve upper extremity function in stroke patients. In particular, tDCS combined with occupational therapy/physical therapy had a significantly greater effect on upper extremity function recovery in stroke survivors with hemiplegia. These results suggest clinicians to consider using tDCS as a compliment to the rehabilitation techniques provided in their clinical settings.

## Figures and Tables

**Table 1:** Characteristics of studies examining the effects of tDCS combined with other rehabilitation therapies on upper extremity function (cont-).

Study	Design	Participants				Interventions	Comparison	Rehabilitation	Outcome Measure	PEDro
		EXP		CTL						
		Sample Size	Mean Age, Onset	Sample Size	Mean Age, Onset					
Alisar et al. [19]	RCT	16	63.56 352.62 days	16	63.50 442.75 days	D-tDCS + OT	Sham tDCS(30 s) + PT/OT	PT/OT for 60–120 min/day, 5 days/week for 3 consecutive day	UE-FM, BSSR-upper extremity, BSSR-hand	7
Allman et al. [20]	RCT	11	59.5 51.2 months	13	66.8 56.6 months	A-tDCS + RAT	Sham tDCS (1 mA/10 s) + RAT	RAT for 60 min/day for 9 consecutive days	ARAT, WMFT, UE-FM	8
Beaulieu et al. [21]	RCT	7	71 55.4 months	7	66.7 86.3 months	D-tDCS + PRT	Sham tDCS (30 s) + PRT	PRT for 60 min/session, 3 times/week for 4 weeks	WMFT, BBT, MAL, mAS	10
Bornheim et al. [22]	RCT	23	62.48 None	23	63.48 None	A-tDCS + PT/OT	Sham tDCS (15 s) + PT/OT	PT/OT for 2 h/day, 5 days/week for 4 weeks	WMFT, FM-UE	10
Cho et al. [23]	RCT	14	58.3 13.2 months	13	60.4 15.5 months	A-tDCS + MT	MT	MT for 20 min/day, 3 times/week for 6 weeks	UE-FM	4
Dehem et al. [24]	RCT	20	62.73 17.47 months	20	58.1 58.5 months	D-tDCS + RAT	sham tDCS + RAT	RAT for 20 min/day for 7 days	BBT, PPT	7
Figlewski et al. [25]	RCT	22	60 9 months	22	61 7 months	A-tDCS + CIMT	Sham tDCS (1.5 mA/30 s) + CIMT	CIMT for 6 h/day for 9 consecutive days	WMFT	7
Hesse et al. [26]	RCT	A-tDCS: 32, 63.9, 3.4 weeks C-tDCS: 32, 65.4, 3.8 weeks Sham tDCS: 32, 65.6, 3.8 weeks				A-tDCS + RAT C-tDCS + RAT	Sham tDCS + RAT	RAT for 20 min/day, 5 times/week for 6 weeks	UE-FM, BBT	9
Kim et al. [27]	RCT	A-tDCS: 6, 55.3, 34 days C-tDCS: 5, 53.6, 19.4 days Sham tDCS: 7, 62.9, 22.9 days				A-tDCS + OT C-tDCS + OT	Sham tDCS (1 min) + OT	OT for 30 min/day, 5 times/week for 2 weeks	UE-FM	8
Kim. [28]	RCT	15	60.2 12.13 moths	15	60.33 10.93 months	D-tDCS + mCIMT	mCIMT	mCIMT for 5 h/day, 5 days/week for 4 weeks	UE-FM, MAL, accelerometer	10
Koh et al. [29]	RCT	14	55.3 15.8 months	11	56.9 13.4 months	tDCS + SM	Sham-tDCS (30 s) + SM	SM for 30 min/day, 3 times/week for 8 weeks	UE-FM, ARAT	9
Lee et al. [30]	RCT	12	None, 6 months	12	None, 6 months	A-tDCS + PT	PT	PT 5 times/week for 4 weeks	UE-FM	4
Lee et al. [31]	RCT	C-tDCS + VRT:20, 63.1, 17.8 days C-tDCS: 19, 60.3, 17.4 days VRT: 20, 60.6, 16.9 days				C-tDCS + VRT	C-tDCS, VRT	VRT for 30 min/day, 5 times/week for 3 weeks	UE-FM, MFT, BBT	7
Lindenberg et al. [32]	RCT	10, 50.3, 20.3 months				tDCS + PT/OT	sham tDCS + PT/OT	PT/OT for 60 min/day for 5 consecutive days	UE-FM, WMFT	9
Lindenberg et al. [33]	RCT	10	61.7 30.5 months	10	55.8 40.3 months	D-tDCS + PT/OT	Sham tDCS (1.5 mA/30 s) + PT/OT	PT/OT for 60 min/day for 5 consecutive days	WMFT, UE-FM	7
Mazzoleni et al. [34]	RCT	12	70 26.6 days	12	75.3 24.2 days	A-tDCS + RAT	Sham tDCS (5 s) + RAT	RAT for 30 min/day, 5 times/week for 6 weeks	UE-FM, BBT	5
Menezes et al. [35]	RCT	22, 56.6, 5.7 years				tDCS + RPSS, sham tDCS + RPSS tDCS + shamRPSS,sham tDCS + shamRPSS		RPSS for 2 h/day	Jamar dynamometer	9
Mortensen et al. [36]	RCT	8	65.5 32 months	8	60.8 28.5 months	A-tDCS + OT	Sham-tDCS (30 s) + OT	OT for 30 min/day for 5 consecutive days	JTT	7
Nair et al. [37]	RCT	7	61 33 months	7	56 28 months	C-tDCS + OT	Sham tDCS + OT	OT for 60 min/day for 5 consecutive days	UE-FM	7
Rabadi et al. [38]	RCT	8	62 6.9 days	8	63 5.9 days	C-tDCS + OT	Sham-tDCS (30 s) + OT	OT for 30 min/day, 5 times/week	ARAT	8
Rocha et al. [39]	RCT	A-tDCS: 7, 58.3, 27.5 months C-tDCS: 7, 58.5, 34.2 months Sham tDCS: 7, 58.5, 26.5 months				A-tDCS + CIMT C-tDCS + CIMT	Sham (30 s)-tDCS + CIMT	CIMT for 6 h/day for 4 consecutive weeks	UE-FM	7
Sattler et al. [40]	RCT	10	67.6 5.3 days	10	62.7 5.6 days	A-tDCS + rPNS	Sham tDCS (1 min) + rPNS	rPNS for 13 min/day for 5 consecutive days	JTT, UE-FM, Hand dynamometer 9HPT	9
Salazar et al. [41]	RCT	15	60 21 months	15	56 23 months	D-tDCS + FES	Sham tDCS(30 s) + FES	FES for 30 min/day, 5 times/week for 2 weeks	A synchronized optoelectronic system, UE-FM, handgrop strength	10
Straudi et al. [42]	RCT	12	52.7 40.7 weeks	11	64.3 78.2 weeks	D-tDCS + RAT	Sham-tDCS (30 s) + RAT	RAT for 30 min/day, 5 times/week for 2 weeks	UE-FM	8
Triccas et al. [43]	RCT	12	64.3 25.3 months	11	62.5 13.4 months	A-tDCS + RAT	Sham-tDCS (10 s) + RAT	RAT for 60 min/day, 2–3 times/week for 8 weeks	UE-FM, ARAT	6
Viana et al. [44]	RCT	10	56 31.9 months	10	55 35 months	A-tDCS + VRT	Sham-tDCS (10 s) + VRT	VRT for 60 min/day, 3 times/week for 5 weeks	UE-FM, WMFT	9
Yao et al. [45]	RCT	20	63 60.5 days	20	66.2 56.5 days	C-tDCS + VRT	Sham-tDCS + VRT	VRT for 20 min/day, 5 sessions/week for 2 weeks	UE-FM, ARAT	8

9HPT: 9 hole pegboard test; ARAT: action research arm test; A-tDCS: anodal transcranial direct current stimulation; BBT: box and block Test; BSSR: brunnstrom stages of stroke recovery; CIMT: constraint-induced movement therapy; C-tDCS: cathodal transcranial direct current stimulation; CTL: control group; D-tDCS: dual transcranial direct current stimulation; EXP: experimental group; FES: functional electrical stimulation; JTT: jebsen-taylor test; mA: milliampere; MAL: Motor Activity Log; mAS = modified Ashworth scale; MFT: manual function test; MT: mirror therapy; OT: occupational therapy; PPT: perdue pegboard test; PT: physical therapy; RAT: robot-assisted therapy; RCT: randomized controlled trials; rPNS: repetitive peripheral nerve stimulation; RPSS: repetitive peripheral nerve sensory stimulation; SM: sensory modulation; UE-FM: upper extremity fugl-meyer score; VRT: virtual reality therapy; WMFT: wolf motor function test; PEDro: Physiotherapy Evidence Database; mCIMT: modified constraint-induced movement therapy.

**Table 2:** Applied transcranial direct current stimulation (t-DCS) parameters for upper extremity function recovery in patients with stroke.

Study	Site	Intensity (mA)	Duration (min.)	A/C/D	Electrode Size (cm^2^)
Alisar et al. [19]	A = C3 of the ipsilesional hemisphere	2	30	D	22
	C = C4 of the contralesional hemisphere				
Allman et al. [20]	A = primary motor cortex (M1) of the affected hemisphere	1	20	A	35
	R = Contralateral supraorbital region				
Beaulieu et al. [21]	A = Ipsilesional M1	2	20	D	35
	C = Contralesionanl M1				
Bornheim et al. [22]	A = MI of the lesioned side	1	20	A	25
	C = Contralesional eye				
Cho et al. [23]	A = M1 of the affected hemisphere	2	20	A	35
	C = Contralateral supraorbital region				
Dehem et al. [24]	A = M1 of the affected hemisphere	1	20	D	35
	C = M1 of the unaffected hemisphere				
Figlewski et al. [25]	A = M1 of the affected hemisphere	1.5	30	A	35
	C = Contralateral supraorbital region				
Hesse et al. [26]	A (A = M1 of the affected hemisphere	2	20	A/C	35
	C = Contralateral supraorbital region)				
	C (C = M1 of the unaffected hemisphere				
	A = Contralateral supraorbital region)				
Kim et al. [27]	A (A = M1 of the affected hemisphere	2	20	A/C	25
	C = Contralateral supraorbital region)				
	C (C = M1 of the unaffected emisphere				
	A = Contralateral supraorbital region)				
Kim. [28]	A = M1 of the affected hemisphere	1	20	D	-
	C = M1 of the unaffected hemisphere				
Koh et al. [29]	A = M1 of the affected hemisphere	1.5	30	D	25
	C = M1 of the unaffected hemisphere				
Lee et al. [30]	C = Non-affected motor region	2	20	C	25
	A = Contralateral supraorbital region				
Lindenberg et al. [32]	A = M1 of the affected hemisphere	1.5	30	D	-
	C = Contralateral M1 area				
Lindenberg et al. [33]	A = M1 of the affected hemisphere	1.5	30	D	16.3
	C = Contralateral M1 area				
Mazzoleni et al. [34]	A = Presumed hand area of affected hemisphere	2	20	D	35
	C = Contralateral orbit region				
Menezes et al. [35]	A = M1 of the affected hemisphere	1	20	A	-
	C = Contralateral supraorbital region				
Mortensen et al. [36]	A = M1 of the affected hemisphere	1.5	20	A	35
	C = Contralateral supraorbital region				
Nair et al. [37]	C = Non-affected motor region	1	30	C	-
	R = Contralateral supraorbital region				
Rabadi et al. [38]	A (A = M1 of the affected hemisphere	1	30	A/C	35
	C = Contralateral supraorbital region)				
	C (C = M1 of the unaffected hemisphere				
	A = Contralateral supraorbital region)				
Rocha et al. [39]	A (A = M1 of the affected hemisphere	1	A = 13	A/C	35
	C = Contralateral supraorbital region)				
	C (C = M1 of the unaffected hemisphere		C = 9		
	A = Contralateral supraorbital region)				
Salazar et al. [41]	A = Ipsilesional M1	2	30	D	25
	C = Contralesionanl M1				
Sattler et al. [40]	A= M1 of the affected hemisphere	1.2	13	A	35
	C = Contralateral supraorbital region				
Shaheiwola et al. [46]	A = M1 of the affected hemisphere	2	20	D	25
	C = M1 of the unaffected hemisphere				
Straudi et al. [42]	A = M1 of the affected hemisphere	1	30	D	35
	C = Contralateral M1 area				
Triccas et al. [43]	A = M1 of the affected hemisphere	1	20	A	20
	C = Contralateral supraorbital region				
Viana et al. [44]	A = M1 of the affected hemisphere	20	13	A	35
	C = Contralateral orbit				
Yao et al. [45]	C = M1 of the unaffected hemisphere	2	20	C	35
	R = Contralateral supraorbital region				

A: anodal transcranial direct current stimulation, C: cathodal transcranial direct current stimulation, D: dual transcranial direct current stimulation, mA: milliampere.

**Table 3:** Effect size of tDCS combined with rehabilitation.

Category	Number of Studies	Effect Size			Heterogeneity			
		d	Z	*p*<	Q Value	df (Q)	*p*	I^2^
Random effects analysis								
Constraint-induced movement therapy	3	0.516	1.245	0.213	6.297	2	0.043	68.241
Robot-assisted therapy	6	0.256	1.674	0.094	1.475	5	0.916	0.000
Occupational therapy/physical therapy	8	0.699	4.364	0.000	7.297	7	0.399	4.064
Virtual reality therapy	3	0.510	2.506	0.012	0.659	2	0.719	0.000
Overall	20	0.483	5.340	0.000	19.793	19	0.407	4.009

tDCS: transcranial direct current stimulation.

## Data Availability

Not applicable.

## Appendix A

1. Search strategy for Pubmed:

#1 “random*” [Text Word] OR ((“Random Allocation”[Mesh]) OR “Randomized Controlled Trial” [Publication Type]) OR “Randomized Controlled Trials as Topic”[Mesh]

#2 animals NOT humans

#3 #1 NOT #2

#4 stroke[tiab] OR “Cerebrovascular Accident” [tiab] OR “Cerebrovascular Accidents” [tiab] OR strokes[tiab] OR Hemiparesis[tiab] OR Hemipareses[tiab]

#5 ((((“Stroke”[Mesh]) OR “Cerebrovascular accident”[Mesh]) OR “Brain Ischemia”[Mesh]) OR “Cerebral Infarction”[Mesh]) OR “Intracranial Hemorrhages”[Mesh]

#6 #4 OR #5

#5 (“Transcranial Direct Current Stimulation”[Mesh]) OR tDCS [Text Word] OR Cathodal [Text Word] OR Anodal [Text Word]

#6 ((((“Physical Therapy Modalities”[Mesh]) OR “Exercise Therapy”[Mesh]) OR “Rehabilitation”[Mesh]) OR “Recovery of Function”[Mesh]) OR “Physical Stimulation” [Mesh]

#7 #5 AND #6

#8 (((((upper-limb[tiab]) OR upper-extremity[tiab]) OR upper limb[tiab]) OR upper extremity[tiab]) OR hand[tiab]) OR arm[tiab]

#9 #3 AND #6 AND #7 AND #8.

2. Search strategy for cochrane controlled register of trials:

#1 stroke or “Cerebrovascular Accident” or “Cerebrovascular Accidents” or strokes or Hemiparesis or Hemipareses: ti, ab, kw

#2 MeSH descriptor: [Stroke] explode all trees

#3 MeSH descriptor: [Brain Ischemia] explode all trees

#4 MeSH descriptor: [Cerebral Infarction] explode all trees

#5 MeSH descriptor: [Intracranial Hemorrhages] explode all trees

#6 #1 or #2 or #3 or #4 or #5

#7 MeSH descriptor: [Transcranial Direct Current Stimulation] explode all trees

#8 MeSH descriptor: [Physical Therapy Modalities] explode all trees

#9 MeSH descriptor: [Exercise Therapy] explode all trees

#10 MeSH descriptor: [Rehabilitation] explode all trees

#11 MeSH descriptor: [Recovery of Function] explode all trees

#12 MeSH descriptor: [Physical Stimulation] explode all trees

#13 #8 or #9 or #10 or #11 or #12

#14 #7 and #13

#15 (upper-limb or upper-extremity or”upper limb” or “upper extremity” or hand or arm): ti,ab,kw

#16 #6 and #14 and #15.

3. Search strategy for EMBASE:

#1 ‘random*’:ab,ti OR ‘randomized controlled trial’:ab,ti OR ‘randomized controlled trial (topic)’:ab,ti OR ‘random allocation’:ab,ti

#2 ‘stroke’:ab,ti OR ‘cerebrovascular accident’:ab,ti OR ‘cerebrovascular accidents’:ab,ti OR ‘strokes’:ab,ti OR ‘hemiparesis’:ab,ti OR ‘hemipareses’:ab,ti

#3 ‘transcranial direct curent stimulation’:ab,ti OR ‘tdcs’:ab,ti OR ‘cathodal’:ab,ti OR ‘anodal’:ab,ti

#4 ‘physical therapy modalities’:ab,ti OR ‘exercise therapy’:ab,ti OR ‘rehabilitation’:ab,ti OR ‘recovery of function’:ab,ti OR ‘physical stimulation’:ab,ti

#5 ‘upper-limb’:ab,ti OR ‘upper-extremity’:ab,ti OR ‘upper limb’:ab,ti OR ‘upper extremity’:ab,ti OR ‘hand’:ab,ti OR ‘arm’:ab,ti

#6 #1 AND #2 AND #3 AND #4 AND #5

4. Search strategy for SCOPUS:

#1 TITLE-ABS-KEY (“random*” OR “Random Allocation” OR “Randomized Controlled Trial” OR “Randomized Controlled Trials as Topic”)

#2 TITLE-ABS-KEY (stroke OR “Cerebrovascular Accident” OR “Cerebrovascular Accidents” OR strokes OR hemiparesis OR hemipareses)

#3 TITLE-ABS-KEY (“Transcranial Direct Current Stimulation” OR tdcs OR cathodal OR anodal)

#4 TITLE-ABS-KEY (“Physical Therapy Modalities” OR “Exercise Therapy” OR “Rehabilitation” OR “Recovery of Function” OR “Physical Stimulation”)

#5 TITLE-ABS-KEY (upper-limb OR upper-extremity OR “upper limb” OR “upper extremity” OR hand OR arm).
